# Selenium Enhances Cadmium Accumulation Capability in Two Mustard Family Species—*Brassica napus* and *B. juncea*

**DOI:** 10.3390/plants9070904

**Published:** 2020-07-17

**Authors:** Zhong-Wei Zhang, Yi-Ying Dong, Ling-Yang Feng, Zong-Lin Deng, Qiang Xu, Qi Tao, Chang-Quan Wang, Yang-Er Chen, Ming Yuan, Shu Yuan

**Affiliations:** 1 College of Life Science, Sichuan Agricultural University, Chengdu 611130, China; zzwzhang@126.com (Z.-W.Z.); dongyiying1120@163.com (Y.-Y.D.); dengzonglin111@163.com (Z.-L.D.); sicauqiangxu@163.com (Q.X.); taoqvip@163.com (Q.T.); zzwzhang@sicau.edu.cn (C.-Q.W.); 2 College of Agronomy, Sichuan Agricultural University, Chengdu 611130, China; fgazelle@126.com; 3 College of Life Science, Sichuan Agricultural University, Ya’an 625014, China; anty9826@163.com (Y.-E.C.); yuanmingsicau@126.com (M.Y.)

**Keywords:** Selenium, chlorophyll fluorescence, Cadmium, subcellular distribution, accumulation capacity

## Abstract

Oilseed rape (*Brassica napus*) is a Cadmium (Cd) hyperaccumulator. However, high-level Cd at the early seedling stage seriously arrests the growth of rape, which limits its applications. *Brassica juncea* had higher Cd accumulation capacity, but its biomass was lower, also limiting its applications. Previous studies have confirmed that Selenium (Se) can alleviate Cd toxicity. However, the regulatory mechanism of Se in different valence states of Cd accumulation was unclear. In this study, we investigated the ameliorating effects of three Se valence states, Na_2_SeO_4_ [Se(VI)], Na_2_SeO_3_ [Se(IV)] and Se-Met [Se(II)], to Cd toxicity by physiological and biochemical approaches in hydroponically-cultured *Brassica juncea* and *Brassica napus* seedlings. Although Se treatments slightly inhibited seedling Cd concentration, it tripled or quadrupled the Cd accumulation level per plant, because dry weight increased about four times more with Se and Cd application than with Cd treatment alone. Among the different valence states of Se, Se(II) had the most marked effect on reducing Cd toxicity as evidenced by decreased growth inhibition and Cd content. The application of Se(II) was effective in reducing Cd-induced reactive oxygen species accumulation, and promoted the antioxidant enzyme activity and photosynthesis of both *Brassica* species. In addition, Se(II) treatment increased the concentrations of Cd in the cell wall and soluble fractions, but the Cd concentration in the organelle part was reduced.

## 1. Introduction

Cadmium (Cd) is a trace heavy metal naturally present in soil. With the development of industries such as mining and smelting, Cd pollution has become increasingly serious [1]. Cd, a non-essential element of plants, has become a common pollutant in farmland soil environment. Cd ions in the soil are absorbed by the plant roots and transported to the leaves via symplast and apoplast transport [2]. Taken up in non-tolerable amounts, Cd causes growth inhibition and even death by deteriorating physiological processes, including biomass production, nutrient acquisition, antioxidant enzyme activities, and photosynthesis [3,4]. Appropriate fertilization, vacuolar sequestration, and adding exogenous ions might be effective in reducing Cd uptake and its accumulation in crops [5,6]. Recent studies show that the addition of Selenium (Se) as an exogenous ion can reduce Cd toxicity in plants.

Selenium (Se) is one of the essential micronutrients for humans and animals, but is not a necessary nutrient for plant growth. The right amount of Se in plants can inhibit peroxidation, restore the integrity of cell membrane structure and function, change the presence and location of heavy metals, and promote growth development [7,8,9]. Previous reports have shown that Se and Cd in plants are antagonistic, Se can reduce the uptake of Cd in plants [10,11]. Se exists mainly in the form of selenate [Se(VI)], selenite [Se(IV)], and selenide [Se(II)] [12]. However, there have only been a few studies on different valences of Se mitigating the mechanism of Cd toxicity [7,8,9,10,11,12].

Oilseed rape (*Brassica napus* L.) is a Cd hyperaccumulator [13]. Black mustard seed (*Brassica juncea* L.) has a strong absorption and accumulation ability for Cd, but its geographical distribution range is limited [14]. Oilseed rape has a wide planting area, high yield, and high economic value, but low absorption capacity for Cd [15]. Thus, we selected *Brassica napus* (BN) and *Brassica juncea* (BJ) as experimental materials. The purpose of this study is to analyze and evaluate different valencies of Se on Cd accumulation in the two *Brassica* species. Cadmium stress at the early seedling stage seriously arrested the growth of rape. Although Se treatments have slight inhibitions on the concentration of cadmium per dry weight, selenium significantly increased the biomass of rape, and, hence, it can triple or quadruple the cadmium accumulation level per plant.

## 2. Results

### 2.1. Effect of Different Valence States of Se on Plant Growth

**Figure 1:**
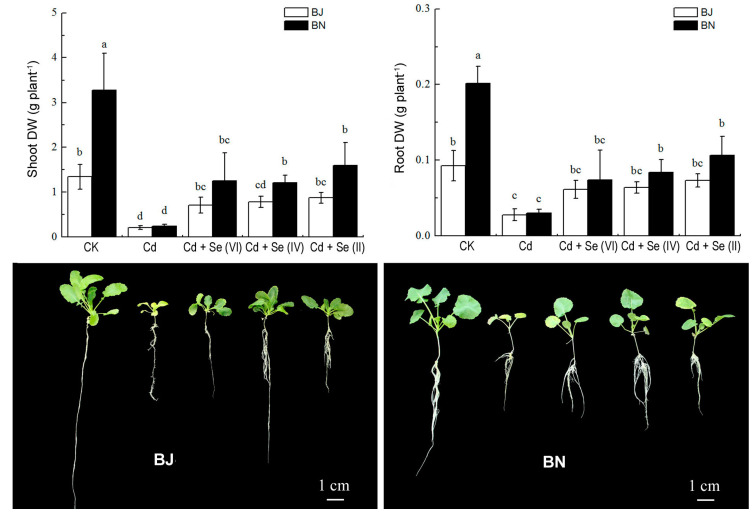
Dry mass variation in shoot and root of *Brassica* seedlings were exposed to five treatments (1) control, (2) 50 μmol/L Cadmium (Cd), (3) 3 μmol/L Na_2_SeO_4_ + 50 μmol/L Cd, (4) 3 μmol/L Na_2_SeO_3_ + 50 μmol/L Cd, (5) 3 μmol/L Selenium (Se)-Met + 50 μmol/L Cd. All values were means ± SD (*n* = 3). Different small letters indicate significant difference at *p* < 0.05.

As shown in Figure 1, the biomass of BN is significantly higher than that of BJ in different treatments. Under Cd treatment, BN seedlings showed obvious symptoms of Cd toxicity, such as leaf chlorosis and growth inhibition. However, Cd-induced growth inhibition was dramatically reduced after the application of Se, and the dry weight (DW) increased about one to four times, compared with Cd-alone treatment. Relative to treatment with Se(IV) and Se(VI), the Se(II) treatment showed a more significant increase in DW in the two *Brassica* species. However, there was no significant difference in the effects of Se on Cd-mediated growth inhibition between the BJ and BN.

### 2.2. Effect of Different Valence States of Se on Se and Cd Content

**Figure 2:**
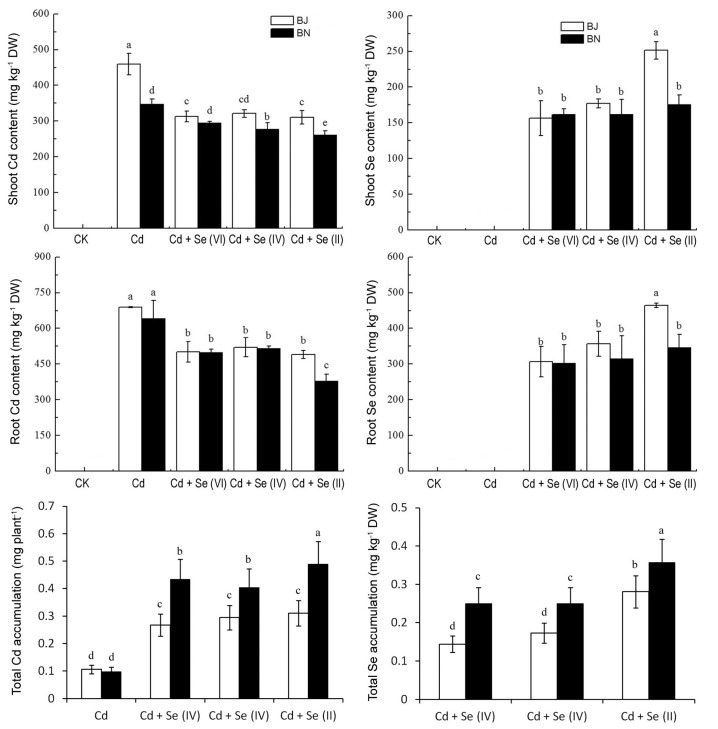
Effects of different treatments on Cd and Se content in the shoot and root of *Brassica* seedlings, DW: dry weight. All values were means ± SD (*n* = 3). Different small letters indicate significant difference at *p* < 0.05.

The accumulation of Cd in BJ was always higher than that in BN, and it can be inferred that absorption of Cd by BJ was stronger than that of BN. The maximum content of Cd was observed in the root and shoot of the plant under Cd-alone treatment. In contrast to Cd-alone treatment, the addition of Se(VI), Se(IV), and Se(II) significantly (*P* < 0.05) decreased Cd levels by 31%, 29%, and 32%, respectively, in the shoot of BJ, but only by 15%, 20%, and 25%, respectively, in the shoot of BN. Therefore, the addition of exogenous Se distinctly reduced the content of Cd, especially Se(II), which could lead to less accumulation of Cd in both BN and BJ seedlings. The Se content in tissues was also below the detection limit when exposed to 50 μM Cd (Figure 2). The Se content in the plant markedly increased after the addition of exogenous Se. BJ accumulation of Se is significantly higher than that BN, the order of Se content was Se(II) > Se(IV) > Se(VI). Although Se treatments slightly inhibited seedling Cd concentration, it tripled or quadrupled the cadmium accumulation level per plant (Figure 2), because the DW increased about four times on Se application compared with that of Cd-alone treatment.

### 2.3. Effect of Different Valence States of Se on H_2_O_2_, O_2_^−^, and Lipid Peroxidation

**Figure 3:**
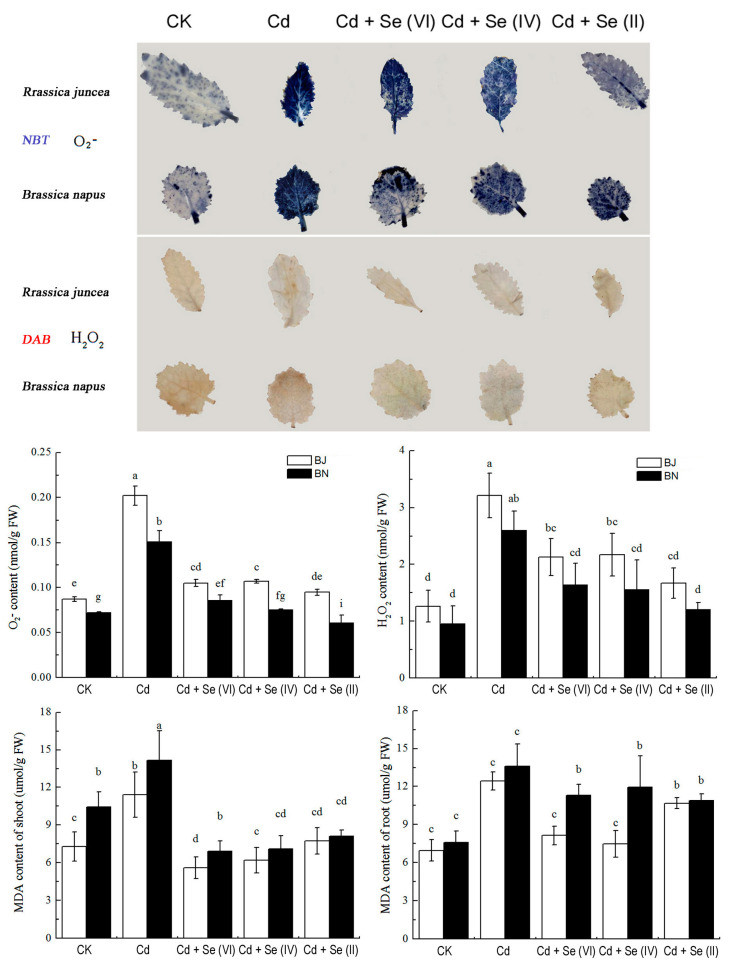
H_2_O_2_ and O_2_^−^ staining in shoot and root after Cd and Se experiment. H_2_O_2_, O_2_^−^, and malondialdehyde (MDA) accumulation in *Brassica* seedlings. FW: fresh weight. All values were means ± SD (*n* = 3). Different small letters indicate significant difference at *p* < 0.05.

Staining with nitro blue tetrazolium (NBT) and 3,3-diaminobenzidine (DAB) dye revealed differences in accumulation of H_2_O_2_ and O_2_^−^ in shoot and quantified throughout the plant (Figure 3). The BN seedlings accumulated more H_2_O_2_ and O_2_^−^ than the BJ. Figure 3 exhibited significant (*p* < 0.05) increase of H_2_O_2_ and O_2_^−^ in Brassica seedlings exposed to 50 μM Cd. However, Se addition significantly reduced (*p* < 0.05) H_2_O_2_ and O_2_^−^∙content. Among them, Se(II) alleviated the Cd-induced H_2_O_2_ and O_2_^−^ accumulation most effectively. Malondialdehyde (MDA) content also showed the same pattern with H_2_O_2_ and O_2_^−^∙content under Cd stress. However, there was no obvious difference of MDA contents in the roots and shoots of *Brassica* seedlings treated with three Se valence states.

### 2.4. Effect of Different Valence States of Se on Antioxidant Enzymes Activities

**Figure 4:**
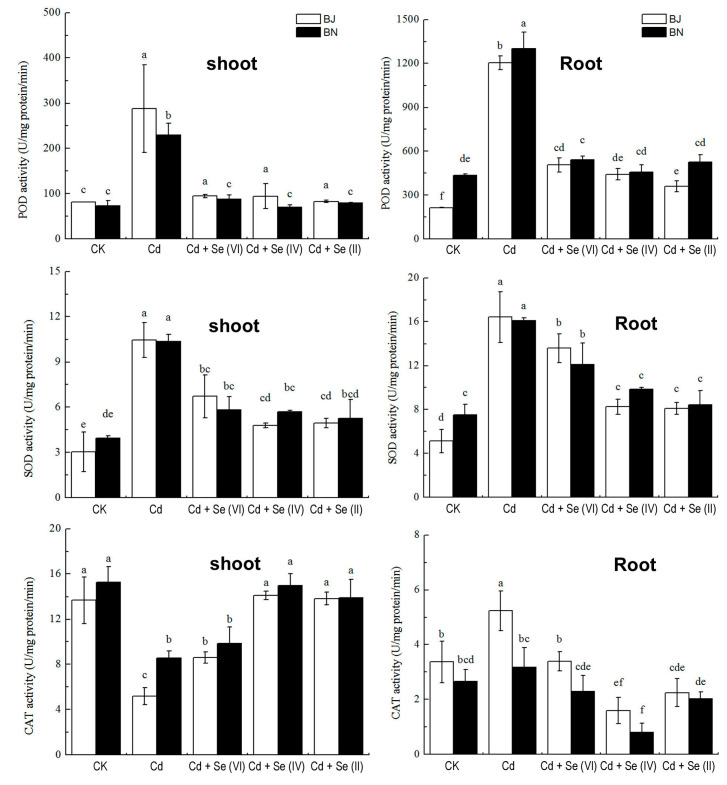
Effects of Cd and exogenous Se application on the superoxide dismutase (SOD), peroxidase (POD) and catalase (CAT) in the shoot and root of two *Brassica* species. All values were means ± SD (*n* = 3). Different small letters indicate significant difference at *p* < 0.05.

Antioxidant enzyme activity exhibited differential responses to Cd stress in shoot and root under different treatments (Figure 4). Cd-alone treatment caused dramatic increases in superoxide dismutase (SOD) and peroxidase (POD) activity either in shoot or root. This increase was significantly reduced by Se supplementation. In terms of catalase (CAT) activity, Se addition was effective in alleviating Cd-inhibited shoot CAT activity. Interestingly, CAT activity was promoted in the BJ, but inhibited in the BN under exposure to Cd alone. However, the change of CAT activity in root caused by Cd was counteracted by the addition of Se. The three valence states of Se, especially Se(II), significantly alleviated the changes in antioxidant enzymes induced by Cd-alone treatment.

### 2.5. Effect of Different Valence States of Se on Photosynthetic Parameters

**Figure 5:**
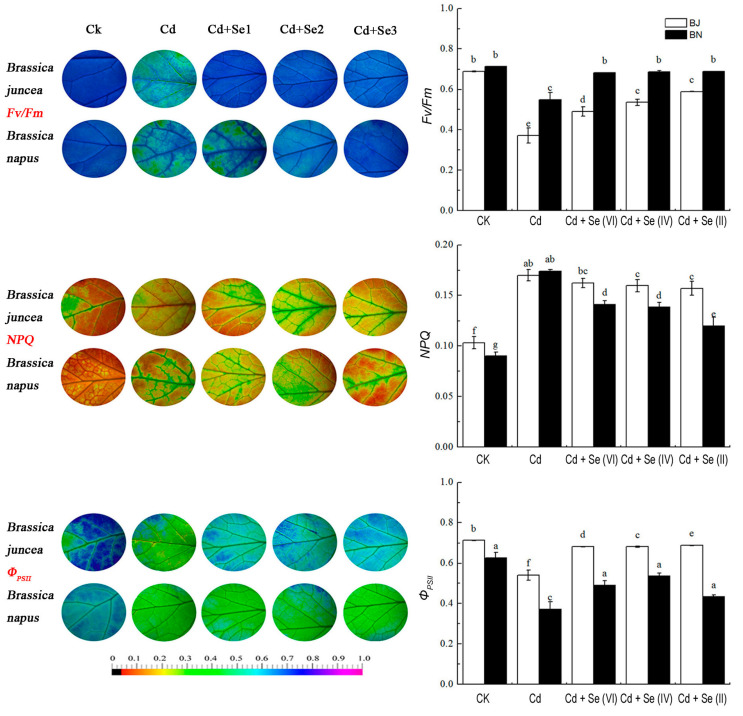
ChlorophyII fluorescence parameters maximum photochemical quantum yield of photosystem II (PSII) *(F*_v_/*F*_m_), actual photochemical efficiency (Φ_PSII_), and non-photochemical quenching (NPQ) of *Brassica juncea* (BJ) and *Brassica napus* (BN) under different treatments. Representative fluorescence images of different treatments are shown on the left panel. Quantitative values are shown on the right panel. *F*_v_/*F*_m_, the maximum efficiency of PSII photochemistry in the dark-adapted state; Φ_PSII_, the quantum yield of PSII electron transport; NPQ, the non-photochemical quenching coefficient. All values were means ± SD (*n* = 3). Different small letters indicate significant difference at *p* < 0.05.

The Cd-alone treatment showed a significant decline in photosynthesis when compared with untreated plants, and the decline was always higher in BJ than BN (Table 1). Both Cd and Se treatment increased the value of net photosynthetic rate (*P*_n_), intercellular carbon dioxide concentration (*C*_i_), and transpiration rate (*T*_r_), but there were no significant differences in Se(II) and Se(VI). Color images visually depicted the changes in maximum photochemical quantum yield of photosystem II (PSII) (*F*_v_/*F*_m_), actual photochemical efficiency (Φ_PSII_), and non-photochemical quenching (NPQ) (Figure 5). The color of the *F*_v_/*F*_m_ image changed from blue to blue with sporadic green in Cd-alone treatment. The leaf color changed from blue to blue-green with sporadic blue in the Φ_PSII_ under Cd stress. The color of the NPQ image changed from red to red (yellow), with sporadic green when exposed to 50 μM Cd. However, the color was recovered markedly after applying the three Se valence states, especially Se(II). The quantified value chart more intuitively reflected the law of *F*_v_/*F*_m_, Φ_PSII_, and NPQ color change in the leaf (Figure 5). The rate of change in BJ was lower than in BN under Cd-alone treatment, indicating that the tolerance of Cd in the two *Brassica* species was different.

### 2.6. Effect of Different Valence States of Se on Subcellular Cd Distribution

**Figure 6:**
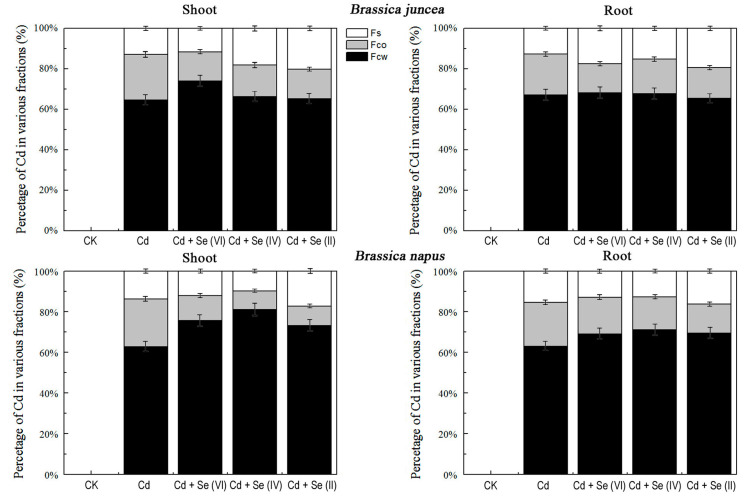
Percentage of Cd content in subcellular distribution of root and shoot in the two *Brassica* species. Fcw, cell wall fraction; Fs soluble fraction, Fco, organelle part fraction. The percentages are mean ± SD (*n* = 3).

We studied the subcellular distribution of Cd to investigate how Se affected the uptake and transport of Cd (Figure 6). A similar pattern was observed in two *Brassica* species under the same treatment. Cd stress significantly increased Cd concentrations in the soluble fraction (Fs) and organelle part fraction (Fco) but reduced that in the cell wall fraction (Fcw). Cd levels in Fcw and Fs were higher than those in Fco in the shoot and root after adding Se in its three valence states (Figure 6). The Cd content of Fco was lowest at Se(II) treatment.

## 3. Discussion

Cadmium ion, Cd^2+^, is highly soluble and migratory and is easily absorbed by plants [1,2]. The biomass production is an important indicator for assessing plant tolerance to Cd toxicity. A study indicated that, although there were no significant differences in the shoot biomass of the Se-supplied plants as compared to the Cd-exposed cucumber plants, the fresh weight of their roots increased significantly after supplementation of a 50 μM Cd-polluted nutrient solution with 10 μM Se [16]. While for the pepper plants at the reproductive stage, Se supplementation improved the flower number, fruit number, and fruit diameter in plants exposed to 0.5 mM Cd [17]. In our study, the exogenous application of Se greatly improved plant growth when exposed to Cd stress (Figure 1). The reason was that an increase in Se can promote carbohydrate metabolism and improve soluble sugar and starch content [18,19]. In addition, Se can combine with other heavy metals to form insoluble complexes, inhibit the absorption of heavy metals by plants, and reduce the accumulation of heavy metals in plants [20]. The addition of Exogenous Se treatment can reduce the risk of Cd-induced oxidative damages in rice and cabbage [11,21]. Similar results were observed in this study; notably, there was a difference in the amount of Cd accumulated under the three valence states of Se treatment (Figure 2).

The lowest Cd content appeared in the Se(II) treatment, which may be related to the absorption of Se. On the one hand, organic Se [Se(II)] was better for plant absorption than inorganic Se [Se(VI), Se(IV)] [22]. After Se(VI) and Se(IV) are absorbed by plants, they are transported via sulfate transporters, and often transformed into selenide (Se(II)) and incorporated into protein [23,24,25,26]. The levels of Se and Cd were higher and lower, respectively, under Se(II) treatment relative to Se(VI) and Se(IV) treatment, which suggests the protective role of Se against Cd toxicity might be dependent on the competition between Se and the heavy metals. On the other hand, plants have evolved mechanisms, such as cell wall compartmentalization, to alleviate heavy metal stress [27,28]. The cell wall is the first barrier to confine the spread of heavy metals into the cell and contains proteins, polysaccharides, and organic acids that effectively bind heavy metal ions, reducing their entry into the protoplast [29,30]. Se(II) can improve Cd binding to the cell wall by increasing its contents of pectin and hemicellulose 2 [31]. Furthermore, Se(II) treatment increased Cd content in the soluble fraction, which may be related to chelation of excess metals with cytosolic ligands (Figure 6). Most Cd in plants is combined with sulfur in sulfur-containing organisms. Se and sulfur are both oxygen-group elements, and have similar chemical properties [32]. Therefore, Se may combine with Cd by replacing sulfur to form a less mobile complex that is accessible to occlusion in the vacuole, thereby inhibiting Cd’s mobility [33]. This might suggest that Se(II) can alleviate Cd toxicity by altering the subcellular distribution of Cd. However, there is no significant difference in the mitigation effect of Se on Cd between the two *Brassica* species.

Under Cd stress, the redox balance in plant cells is destroyed and large amounts of ROS accumulate, which produces oxidative stress [14]. The exogenous addition of Se significantly inhibited Cd-induced oxidative stress by the increase in the concentrations of H_2_O_2_, O_2_^−^, and MDA (Figure 3). Similar evidence that Se played a favorable role in inhibiting plant oxidative stress under Cd stress has been observed previously [34,35]. Se(II) treatment significantly counteracted the increase in H_2_O_2_ and O_2_^−^ levels induced by Cd, compared to treatments of Se(VI) and Se(IV) (Figure 3). This may be related to the different amounts of Cd accumulation in *Brassica* seedlings. In addition, plants develop enzymatic systems to scavenge ROS in cells and oxidative damage, maintaining the redox balance under Cd stress [36]. Se(II) treatment can catalyze O_2_^−^∙disproportionation to produce H_2_O_2_, which can be further degraded to completely non-toxic H_2_O and O_2_ to defend against cell membrane lipid peroxidation [37]. Figure 4 provides strong evidence that different Se treatments (especially Se(II) treatment) could effectively counteract the changes of Cd-induced antioxidant enzymes [38,39].

Chlorophyll fluorescence and gas exchange are considered to be important indicators of plant response to heavy metal stress [40,41,42]. Under Cd-alone treatment, photoinhibition of *Brassica* plants occurs, causing the reaction center of PSII to be inactivated or destroyed, thereby inhibiting photosynthesis. Se can alleviate Cd-mediated inhibition on photosynthesis of two *Brassica* species, especially Se(II) (Table 1). This may be related to Se(II), as Se(II) can increase antioxidant capacity of some enzymes to enhance the plant’s resistance to Cd stress [43]. In addition, toxic metals damage the antenna molecules and diminish the chlorophyll fluorescence [42,44]. PSII’s overall photosynthetic capacity was diminished under Cd-alone treatment in this study, but supplementation of Se reduced the Cd-induced disruption in photosynthetic apparatus. After Se supplementation, *Brassica* seedlings enable non-optical energy consumption for self-protection and alleviate Cd-induced damage to photosynthesis. Consistent with this, Se supplementation could improve Cd-induced chlorophyll fluorescence in mustard and tomato [45,46].

## 4. Materials and Methods

### 4.1. Plant Materials and Experimental Design

Seeds of *Brassica napus* (cultivar Zhongshuang 11) and *Brassica juncea* (cultivar Yongbao 2) were sterilized, and vernalized with 2 mg/L of gibberellin at 4 °C for 3 days. The seeds germinated on moist filter paper in the dark at 25 °C for 3–4 days, and were sown in culture medium (1/2 Hoagland nutrient solution with 0.25% agar powder) in an artificial controlled chamber with a 16 h day/8 h night at 25 °C/18 °C cycle, relative humidity 70%, and light intensity of 100 μmol m^−2^ s^−1^. When the true leaf was fully expanded, similar size seedlings were transplanted to a plastic container and cultured in 1/2 Hoagland-Arnon nutrient solution.

On two weeks after transplanting, we applied CdCl_2_, Na_2_SeO_4_ [Se(VI)], Na_2_SeO_3_ [Se(IV)] and Se-Met [Se(II)] to the corresponding containers: (1) Control (CK: without Cd or Se); (2) Cd, 50 μmol/L CdCl_2_; (3) Cd + Se(VI), 3 μmol/L Na_2_SeO_4_ + 50 μmol/L CdCl_2_; (4) Cd + Se(IV), 3 μmol/L Na_2_SeO_3_ + 50 μmol/L CdCl_2_; (5) Cd + Se(II), 3 μmol/L Se-Met + 50 μmol/L CdCl_2_. Plant samples were collected after two weeks of treatment and were used for various biochemical assays (each with four replicates).

### 4.2. Plant Growth, Biomass, and Element Determination

The shoot and root were separated and washed thoroughly with deionized water, and then the samples were oven-dried at 75 °C for 2 days until constant weight was achieved, to determine the DWs of the shoot and the root. The shoot and root of *Brassica* seedlings that have been baked to constant weights were ground into a powder. Approximately 0.3 g samples were digested by a microwave system (MWD-500, Metash instrument Co., Ltd., Shanghai, China) in a mixed acid containing HNO_3_/HClO_4_ (v/v:4/1), and then sequentially diluted to 50 mL. Concentrations of Cd and Se were determined using inductively coupled plasma-mass spectrometry (ICP-MS) (7900, Agilent, Santa Clara, CA, USA).

### 4.3. Histochemical Staining and Determination of H_2_O_2_, O_2_^−^, and Lipid Peroxidation

Nitro blue tetrazolium (NBT) and 3,3-diaminobenzidine (DAB) were used to examine visible superoxide (O_2_^−^) and hydrogen peroxide (H_2_O_2_) in plant leaves [47]. After sampling, leaves were stained with NBT (0.5 mg/mL) and DAB (2 mg/mL) for 2 h and 8 h, respectively. Leaves were bleached in boiling 95% ethanol. Superoxide content was quantitated, as described by Chen et al. [39]. H_2_O_2_ content was quantitated by the hydroxylamine method [41]. Malondialdehyde (MDA) content was determined using thiobarbituric acid-reactive substances (TBARS) [48].

### 4.4. Determination of Antioxidant Enzyme Activity

The fresh samples were homogenized with 5 mL of extractant [50 mM Tris-HCl (pH 7.0) with 20% glycerin, 1 mM of ascorbic acid (AsA), 1 mM of dithiothreitol (DTT), and 1 mM of glutathione (GSH)] using pre-chilled mortar and pestle. The homogenate was centrifuged at 20,000× *g* for 20 min at 4 °C, and the supernatant was collected for testing. Superoxide dismutase (SOD) activity was determined by nitroblue tetrazolium [49]; catalase (CAT) activity was determined by UV spectrometry method [50]; and peroxidase (POD) activity was determined by guaiacol method [51].

### 4.5. Determination of Photosystem Parameters and Chlorophyll Fluorescence

Photosynthetic indicators were determined using a portable photosynthetic apparatus (LI-6400; LICOR, Lincoln, NE, USA), and the measurement times were selected from 9:00 am to 11:00 am. The measurement parameters included net photosynthetic rate (*P*_n_), intercellular carbon dioxide concentration (*C*_i_), stomatal conductance (*G*_s_), and transpiration rate (*T*_r_).

The treated *Brassica* seedlings were placed in the dark for 30 min. The fluorescence parameters of *Brassica* leaves were determined by IMAGING-PAM (Heinz-Walz Instruments, Effeltrich, Germany) at room temperature. The parameters determined in this study included maximum photochemical quantum yield of PSII (*F*_v_/*F*_m_), actual photochemical efficiency (Φ_PSII_), and non-photochemical quenching (NPQ).

### 4.6. Subcellular Distribution of Cd

Gradient centrifugation technique was used for subcellular separation of Cd [52]. The fresh sample tissues were ground in a 50 mM Tris-HCL buffer (pH 7.5) containing 0.25 M of sucrose and 1.0 mM dithioerythritol. The supernatant was centrifuged at 3000× *g* for 15 min, and the retained pellet was the cell wall fraction (Fcw). The supernatant solution was further centrifuged (12,000× *g*, 30 min), and the resultant deposition was the organelle-containing fraction (Fco). The remaining supernatant was the soluble fraction (Fs). The metal concentrations were determined as described above.

### 4.7. Data Analysis

The data were statistically analyzed by Duncan test with SPSS 20.0 (IBM Research, New York, NY, USA), and all diagrams were drawn using OriginPro 8 (OriginLab, Northampton, MA, USA). The data were the average of the three replicates.

## 5. Conclusions

Although Se treatments slightly reduced Cd concentrations in *Brassica* seedling, they tripled or quadrupled the Cd accumulation level per plant, because the dry weight increased about four times more under Se + Cd co-treatment than under Cd treatment alone. Among the different valence states of Se, Se(II) had the most marked effect on alleviating Cd toxicity, as evidenced by promoting growth and inhibiting Cd accumulation. The application of Se(II) was effective in reducing Cd-induced reactive oxygen species accumulation, and promoted the antioxidant enzyme activities and photosynthetic activities. At the subcellular level, Se(II) treatment increased the concentrations of Cd in the cell wall and soluble fractions, but decreased Cd level in the organelle fraction.

## Figures and Tables

**Table 1:** Photosynthesis of two *Brassica* species under the different treatments. Values are mean ± SD (*n* = 3). Different small letters indicate significant difference at *p* < 0.05.

Materials	Treatments	*P*_n_ (μmol m^−2^ s^−1^)	*G*_s_ (mmol m^−2^ s^−1^)	*C*_i_ (mmol m^−2^ s^−1^)	*T*_r_ (μmol m^−2^ s^−1^)
BJ	CK	14.36 ± 0.03b	1.08 ± 0.08a	399.68 ± 1.51e	1.45 ± 0.03d
	Cd	6.59 ± 0.01i	0.16 ± 0.00f	374.63 ± 0.50f	0.77 ± 0.00g
	Cd + Se(VI)	9.37 ± 0.06g	0.68 ± 0.01d	434.11 ± 0.06c	2.54 ± 0.02a
	Cd + Se(IV)	12.95 ± 0.06d	0.77 ± 0.05c	349.80 ± 1.50h	2.18 ± 0.04d
	Cd + Se(II)	13.32 ± 0.00c	0.49 ± 0.01e	373.08 ± 0.42fg	1.99 ± 0.00c
BN	CK	16.85 ± 0.02a	0.04 ± 0.00e	442.03 ± 0.12b	2.20 ± 0.00b
	Cd	5.77 ± 0.01j	0.12 ± 0.01f	400.25 ± 2.42e	0.24 ± 0.02h
	Cd + Se(VI)	8.89 ± 0.06h	0.96 ± 0.01b	423.74 ± 0.37d	1.32 ± 0.00e
	Cd + Se(IV)	11.49 ± 0.09f	0.14 ± 0.00f	371.07 ± 2.41g	1.22 ± 0.02f
	Cd + Se(II)	12.00 ± 0.04e	0.73 ± 0.00c	492.57 ± 0.14a	1.97 ± 0.00c

*P*_n_, net photosynthetic rate; *G*_s_, stomatal conductance; *C*_i_, intercellular carbon dioxide concentration; *T*_r_, transpiration rate.

## Abbreviations

AsA	ascorbic acid
BJ	*Brassica juncea*
BN	*Brassica napus*
CAT	catalase
Cd	Cadmium
*C*_i_	intercellular carbon dioxide concentration
DAB	3,3-diaminobenzidine
DTT	dithiothreitol
DW	dry weight
Fco	organelle part fraction
Fcw	cell wall fraction
Fs	soluble fraction
*F*_v_/*F*_m_	maximum photochemical quantum yield of PSII
*G*_s_	stomatal conductance
GSH	glutathione
ICP-MS	inductively coupled plasma-mass spectrometry
MDA	malondialdehyde
NBT	nitro blue tetrazolium
NPQ	non-photochemical quenching
*P*_n_	net photosynthetic rate
POD	peroxidase
PSII	photosystem II
Se	Selenium
SOD	superoxide dismutase
TBARS	thiobarbituric acid-reactive substances
*T*_r_	transpiration rate
Φ_PSII_	actual photochemical efficency
